# The Plastid Genome of *Najas flexilis*: Adaptation to Submersed Environments Is Accompanied by the Complete Loss of the NDH Complex in an Aquatic Angiosperm

**DOI:** 10.1371/journal.pone.0068591

**Published:** 2013-07-04

**Authors:** Elena L. Peredo, Ursula M. King, Donald H. Les

**Affiliations:** Department of Ecology and Evolutionary Biology, University of Connecticut, Storrs, Connecticut, United States of America; University of Nottingham, United Kingdom

## Abstract

The re-colonization of aquatic habitats by angiosperms has presented a difficult challenge to plants whose long evolutionary history primarily reflects adaptations to terrestrial conditions. Many aquatics must complete vital stages of their life cycle on the water surface by means of floating or emergent leaves and flowers. Only a few species, mainly within the order Alismatales, are able to complete all aspects of their life cycle including pollination, entirely underwater. Water-pollinated Alismatales include seagrasses and water nymphs (*Najas*), the latter being the only freshwater genus in the family Hydrocharitaceae with subsurface water-pollination. We have determined the complete nucleotide sequence of the plastid genome of *Najas flexilis*. The plastid genome of *N. flexilis* is a circular AT-rich DNA molecule of 156 kb, which displays a quadripartite structure with two inverted repeats (IR) separating the large single copy (LSC) from the small single copy (SSC) regions. In *N. flexilis,* as in other Alismatales, the *rps19* and *trnH* genes are localized in the LSC region instead of within the IR regions as in other monocots. However, the *N. flexilis* plastid genome presents some anomalous modifications. The size of the SSC region is only one third of that reported for closely related species. The number of genes in the plastid is considerably less. Both features are due to loss of the eleven *ndh* genes in the *Najas flexilis* plastid. In angiosperms, the absence of *ndh* genes has been related mainly to the loss of photosynthetic function in parasitic plants. The *ndh* genes encode the NAD(P)H dehydrogenase complex, believed essential in terrestrial environments, where it increases photosynthetic efficiency in variable light intensities. The modified structure of the *N. flexilis* plastid genome suggests that adaptation to submersed environments, where light is scarce, has involved the loss of the NDH complex in at least some photosynthetic angiosperms.

## Introduction

Chloroplasts evolved from prokaryotic photosynthetic endosymbionts [1] as cell organelles that maintain their own genetic material in a double stranded DNA molecule ranging in size from 35 to 217 kb [2]. Compared to their cyanobacterium-like ancestors, plastid genomes have experienced a dramatic reduction in gene number from the +3 000 once present in free-living Cyanobacteria to only 120–250 genes in photosynthetic eukaryotes. The plastid genes that have been retained encode products necessary for photosynthetic and housekeeping functions. During photosynthetic eukaryote evolution, cyanobacterial genes were transferred from the endosymbiont to the host nucleus or were lost entirely, in instances where the function of those genes was no longer essential [3]. The process of gene transfer has not stopped [4] but continues as a constant flood of plastid and mitochondrial genome fragments to the nucleus, where organelle DNA can be integrated as functional genes. However, over time, such genes usually are pseudogenized and lost, with only a small proportion of the transferred DNA integrated into functional areas and being conserved [4]. Red algal plastids retain the highest number of genes of any other group of photosynthetic eukaryotes (232–252) [3]. In contrast, the chloroplast of land plants (Embryophyta), and of their ancestral green algae (Chlorophyta), retains only 120 genes. It usually consists of two copies of an inverted repeat (IRa, IRb) that separate a large single copy region (LSC) from a small single copy region (SSC). While missing in some algae (Glaucophyta, Rhodophyta), green plant plastids are rich in repeated regions and possess editing mechanisms [3].

Key photosynthetic elements are encoded in plastid genomes, such as photosystem I and II genes, RuBisCO and thylakoid NAD(P)H dehydrogenase. Independent of any former function of *ndh* genes in Cyanobacteria, *ndh* genes are essential for photosynthesis in land plants [5]. Lost in other algal divisions, the *ndh* genes probably were essential in the adaptation of green algae to the fluctuating conditions of shoreline environments [5] The eleven plastid *ndh* genes together with four nuclear genes (*nhdL*, *ndhM*, *ndhN*, and *ndhO*) encode the thylakoid NAD(P)H dehydrogenase complex which functions mainly in the electron transfer from NADH to plastoquinone, which protects the cell against photooxidative-related stress and maintains optimal rates of cyclic photophosphorylation [5]. In land plants, small changes in any of the *ndh* genes significantly decrease net photosynthesis [6]. As a consequence of such strong selective pressure, the *ndh* genes are highly conserved across all vascular plant divisions [7].

In angiosperms, *ndh* loss in plastomes is associated primarily with heterotrophic (i.e., parasitic) plants [8]. The plastid genome of non-photosynthetic organisms undergoes severe rearrangements and deletions that lead to losses of both photosynthetic and chlororespiratory genes, which no longer are needed to maintain metabolic functions. Convergent (homoplasious) losses of *ndh* genes are evident among unrelated parasitic plants; such as *Epifagus* (Lamiales) [9], *Cuscuta* (Solanales) [10], [11] or the mycotrophic orchid *Neottia* [12]. Pseudogenization and loss of *ndh* genes in the parasitic bryophyte *Aneura mirabilis* [13] further substantiates the relationship between parasitism and *ndh* loss. Due to the mutual interaction between symbiotic fungi and orchids [14], it is understandable that the lack of functional *ndh* genes is widespread within the Orchidaceae, even green-leaved orchids [15]–[17]. Recent data suggest pseudogenization of *ndhB* and even complete loss of *ndhF* for some taxa in the order Alismatales (seagrasses and water nymphs) [18]. Homoplasic loss of *ndh* genes in aquatic angioperms might be related to potential adaptations to the constraints of the underwater environment [19]. The *ndh* genes are also absent in some photosynthetic gymnosperms [20]. The shared loss of *ndh* genes in Gnetales and Pinaceae is regarded as a rare synapomorphic event, which provides support to the gnepine hypothesis [8]. While the loss of *ndh* genes in Gnetales and Pines occurred early in the evolution of land plants, *ndh* loss in some *Erodium* (Geraniaceae) species provides an example of particularly recent loss [21]. The possible explanation for *ndh* genes loss in Gnetales, pines and some *Erodium* species is difficult to elucidate.

Recolonization of aquatic environments by land plants has occurred up to 100 independent times, comprising up to 2% of the approximate 350,000 angiosperm species [22]. These recolonization events presumably would have required many physiological, metabolic and reproductive adaptations essential to accommodate the broad and novel conditions of aquatic habitats, which range from warm and sunny shallow waters to cool and dark deep waters. The monocot order Alismatales includes ∼4500 extant species in 13 families, which includes several that are predominantly aquatic (see [18], [22] for phylogeny). The order encompass various wetland and aquatic life-forms ranging from emergent, floating-leaved, free-floating and submersed, only the latter life-form fully adapted to the aquatic environment. Additionally, a diverse array of reproductive strategies representing different degrees of adaptation to the aquatic environment is evident and includes anemophily, entomophily, self-pollination, unusual water-facilitated types involving detached, floating flowers, and both surface and subsurface water-pollination [23]. Complete adaptation to submersed life, including water-pollination (hydrophily), is quite rare, being present in only 130 angiosperm species [22]. The greatest concentration of hydrophilous species occurs in the Alismatales, most notably in seagrasses and water nymphs. The genus *Najas L.* (water nymph) includes up to 40 species, all of them completely submersed and hydrophilous. It is the only freshwater genus in the family Hydrocharitaceae with subsurface water-pollination. *Najas* exhibits a number of morphological adaptations associated with the successful colonization of deep water habitats, such as a lack of stomata and reduced leaves formed by only two layers of epidermal cells [24]. However physiological adaptations to maximize underwater photosynthesis have remained obscure.

Arguably, the low light levels found in submersed aquatic environments should increase selective pressures on some features, while reducing the adaptive potential of others. Recent work has indicated that such selection has induced changes even in genes extremely conserved across plant evolution. For example, the 25-nucleotides of the intercistronic spacer region in the photosynthesis-related genes *psaA*-*psaB* is highly conserved through plant evolution, from green algae to land plants. Yet, parasitic plants, where photosynthetic genes are lost or pseudogenized, and aquatics, which are under different selective pressures than terrestrial plants, accumulate most of the variants detected in that plastid region [19]. In particular, the genus *Najas* possesses a unique spacer configuration that likely regulates transcription of these genes in response to temperature [19].

To provide additional information on the plastid genome structure of fully submersed water-pollinated aquatic angiosperms, we have sequenced the complete plastid genome of *Najas flexilis* (Willd.) Rostk. & Schmidt (nodding waternymph). We have analyzed the structure and gene content of the *Najas flexilis* plastid genome and have compared the *Najas flexilis* plastome to those available for other monocots and angiosperm species. We have designed specific primers to evaluate problematic areas such as the reduced SSC region, and investigated their cross-transferability in other *Najas* species. Modifications in the chloroplast of *Najas flexilis* reported here may provide insight on the importance of the plastid NAD(P)H dehydrogenase during land colonization by photosynthetic eukaryotes.

## Materials and Methods

### 1. DNA Extraction and Sequencing

Plant material was collected from two sites that potentially reflect genetic diversity in *Najas flexilis*. *Najas flexilis* ‘canadensis’ was collected in Scotland, Lower Glenastle Loch (Islay, Scotland; 18 August 2010) permit issued by the Scottish Natural Heritage (License number 11012) and *Najas flexilis* ‘flexilis’ was collected in the USA (Connecticut), from the public access site at West Side Pond with permission from the CT DEEP (20 September 2010). Fresh material of each sample was stored in saturated NaCl-CTAB solution [25]. Prior to DNA extraction, leaves of a single plant were washed in double-distilled water and carefully cleaned under a dissecting microscope to remove epiphytes from the plant tissue surface.

Total DNA was extracted using standard procedures [26], re-suspended in 30 µl of 1X TE buffer and checked in a 2% agarose gel. DNA concentration was determined by measuring the optical density at 260 nm and 280 nm in a NanoDrop ND-1000 spectrophotometer (Thermo Scientific, Asheville, NC, USA). Molecular confirmation of the identity of each accession was performed by sequencing the ITS, *trnK*- *matK*, and *rbcL* regions as described previously [23].

Genomic DNA (500 ng) was sheared by nebulization, subjected to 454 library preparation and shotgun sequencing using the Genome Sequencer (GS) FLX Titanium pyrosequencing platform (454 Life Science Corporation, Branford, CT, USA) at the in-house facility (Center for Applied Genetics and Technology) at the University of Connecticut. Two independent runs (1/16 picotiter plate each) were performed with each library to a total of 1/4 picotiter plate for the species. The sequencing runs provided a total of 198 152 raw reads of which 80 858 were assigned successfully to the Scottish material and 116 118 to the American material. Reads were trimmed in CLC Genomics Workbench 5.1 (CLC bio, Aarhus, Denmark) under the following criteria: quality score 0.05, mismatches 2. Reads under 70 nt or over 800 nt were discarded. Average length for each of the runs was 388.5 and 301.9. As the same libraries were used in the second run, this length decay was expected. Over 95% of the reads presented a PHRED score over 25.

### 2. Genome Assembly and Annotation

Out of 191 597 raw reads that were above the quality threshold, 84 739 reads were assembled in 10 979 contigs (aprox 5 mill nt) using CLC Genomic Workbench. Contigs were filtered against the *Lemna minor* complete plastid sequence (NC_010109) [27]. Additional identification of chloroplast contigs was performed using the blastn algorithm in Blast2Go [28]. A total of 51 contigs with an average length of 2 306 nt corresponded to the *Najas flexilis* plastid genome. The longest contig extended to 24.5 kb and included most of the inverted repeat region. Average sequencing depth was 12-fold, reaching maximum numbers in the IR region (44-fold). Total coverage of the combined contigs was 117 603 nucleotides leaving 6% of the plastid genome unresolved when only one IR region was included. Where possible, gaps were closed between *de novo* contigs using consensus sequences of newly assembled reads in Geneious version 5.6 (Biomatters; available from http://www.geneious.com/). To maximize the number of reads assembled the concatenated *Najas flexilis* contigs were used as new references for filtering reads prior to *de novo* re-assemble of contigs.

Sixteen areas of the scaffold were checked by PCR and ABI sequencing (Table S1). These areas included gaps or low coverage areas, and structurally conflicting features, such as junctions and areas in the SSC. Primers were designed using Primer 3 [29] as implemented in Geneious. Duplicate PCR mixes were prepared for each primer set and included 1 U Titanium Taq (Clontech), 1x reaction buffer, 0.2 um of each primer, and 5–15 ng of DNA. One of the reaction mixes included the PCR additive betaine (Affymetrix) to a final concentration of 1 uM. Optimal annealing temperatures for each primer set were tested by gradient PCR (annealing temperature ranging from 52 C to 58 C) for each primer set and PCR mix. At least two PCR reactions were sequenced for each primer combination.

The fully sequenced *Najas flexilis* genome was annotated in Geneious by using the ORF finder plug-in along with comparison to other annotated plastid genomes. Annotations were checked using DOGMA (Dual Organellar GenoMe Annotator) [30] and additional ORF searches were performed with Glimmer 3 [31] and ORF Finder (T. & R. Tatusov) both available through the NCBI website.

### 3. Examination of Genome Structure

Mauve [32] was used to perform multiple genome alignments and explore the large-scale structure of the plastid genome of *Najas flexilis*. Available plastid genomes of species belonging to the Alismatales were aligned with Progressive Mauve algorithm using *Acorus* as an outgroup. Similar alignments were performed using broader phylogenetic groups (i.e., available monocots or angiosperms).

An additional whole-genome analysis was conducted using the CGView Comparison Tool [33]. The plastome of *Najas flexilis* was compared to those of other available angiosperms, using representatives of the major groups spanning the entire angiosperm phylogeny as outlined in [34]. Two different sets of blast maps were generated based on DNA-DNA and CDS analysis. In these maps, the sequence identity was represented by a different color scheme related to the BLAST hits (blastn and blastp). Additional features such as identification of GC rich areas in the reference genome or GC Skew index were also included. As a means of visual comparison, an analysis using the same parameters was also run for the closely related species *Elodea canadensis* (JQ310743) [35].

## Results and Discussion

### 1. *Najas flexilis* Plastid Genome

**Figure 1:**
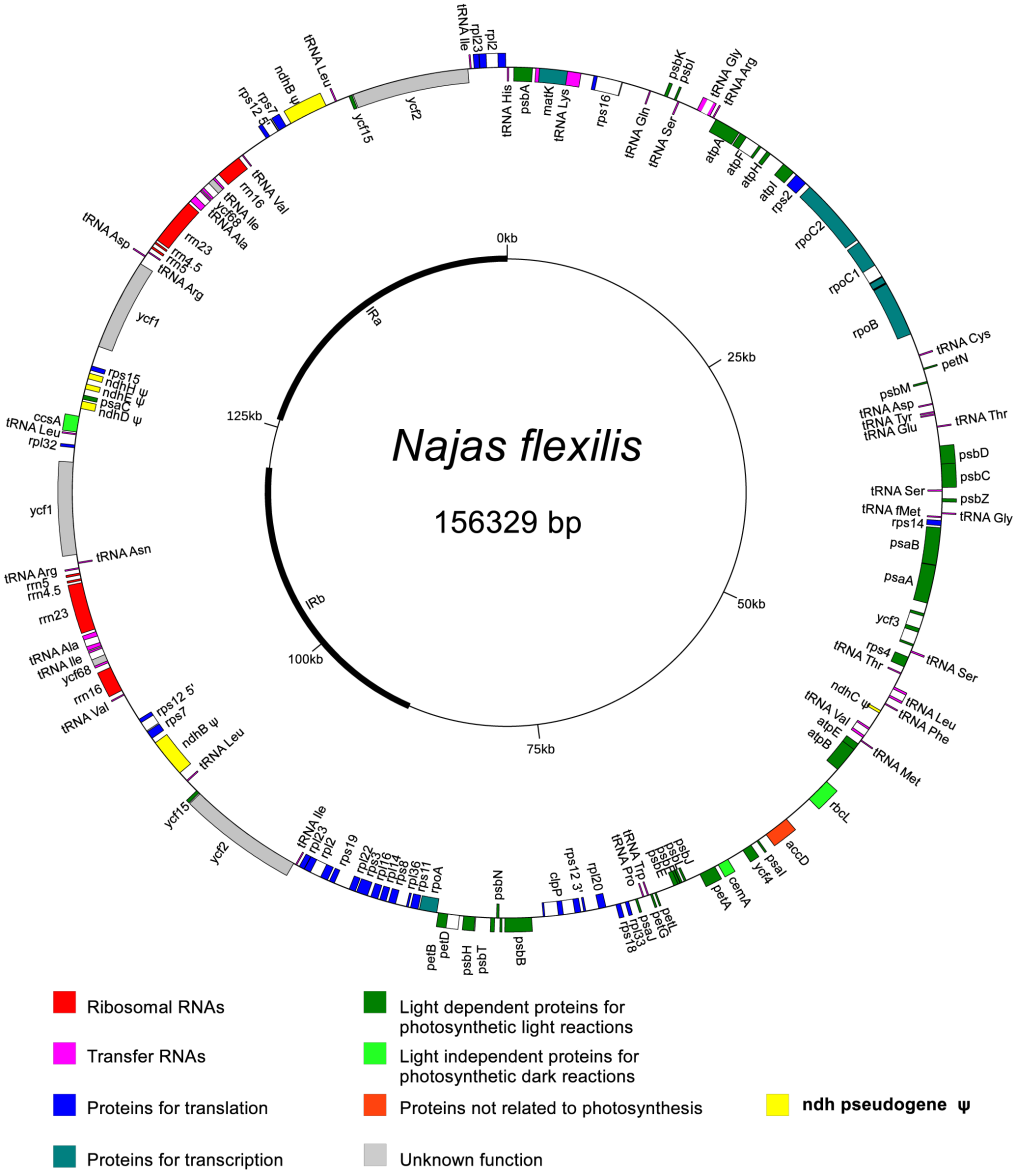
The *Najas flexilis* chloroplast genome. Inner circle, schematic representation of the quadripartite structure of the genome, IR regions outlined in black. Outer circle, gene organization in the genome. Genes shown outside the circle are transcribed counterclockwise; genes in the inside are transcribed clockwise. Genes are color-coded by function.

The determined sequence for the *Najas flexilis* plastid genome is 156 329 bp (Fig. 1, GenBank accession JX978472). It displays a circular quadripartite structure, with the large single copy region (LSC; 88 668 bp, 56.72% of the total genome) separated from the small single copy region (SSC; 5 266 bp, 3.36%) by two inverted repeat regions (IRb and IRa; 31 198 bp, 19.95% each). Average GC content is 38.2%, similar to those reported for such closely related monocots, as *Smilax china* (37.25%) [36], *Elodea canadensis* (37%) [35], and members of the Araceae family (35.2% to 35.9%) [27], [37]. The IR regions encode, among other genes, ribosomal gene cluster and therefore they display a higher GC content than either the small or large single copy areas (42.9% vs. 30.4% and 35.4%).

The plastome of *Najas flexilis* codes for 103 unique genes, sixteen of which are duplicated in the inverted repeat regions (Table 1 and Fig. 1). Seventeen genes present discontinuous reading frames; 14 have a single intron and two genes have two introns. The *rps12* gene is transpliced with exon 1 coded in the LSC region and exons 2 and 3 in the IRs. In the closely related species *Elodea canadensis*, the *rpl16* is interrupted by a 1 kb intron [35] and the two exons expand for 10 and 400 nt respectively. In *Najas flexilis*, the *rpl16* intron is not present. Maximum likelihood trees of the coding region of *rpl16* (nt/aa) successfully placed *Najas flexilis* in the same clade as *Elodea* and separate from the *Spirodela*-*Lemna*-*Wolffia*-*Wolffiella* clade (both 99%–100% bootstrap support, data not shown), showing that neither the sequence nor protein encoded by the *rps16* gene had been altered excessively by the intron loss.

**Table 1:** Genes encoded in the *Najas flexilis* chloroplast.

Function	Gene Class	Genes	Number
**Proteins not related to** **photosynthesis**	Acetyl-CoA carboxylasecarboxyltransferase beta subunit	*accD*	1
**Light independent proteins**	Membrane protein	*cemA*	1
	Cytocrome c biogenesis	*ccsA*	1
	Rubisco	*rbcL*	1
**Light dependent proteins**	ATP synthase	*atpA*, *atpF* *, *atpH*, *atpI*	4
	ndh Genes		
		*ndhB*Ψx2, *ndhC*Ψ, *ndhD*Ψ, *ndhE*Ψ, *ndhH*Ψ	
	Photosystem I assembly factor	*yfc3* **, *ycf4*	2
	Cytocrome	*petA*, *petB* *, *petD* *, *petG*, *petN*, *petL*	6
	Photosystem I	*psaA*, *psaB*, *psaC*, *psaI*, *psaJ*	5
	Photosystem II	*psbA*, *psbB*, *psbC*, *psbD*, *psaE*, *psbF*, *psbH*, *psbI*, *psbJ*, *psbK*, *psbL*, *psbM*, *psbN*, *psbT*, *psbZ*	15
**Genetic apparatus**	Maturase	*matK*	1
Proteins for transcription	RNA polymerase	*rpoA*, *rpoB*, *rpoC1* *, *rpoC2*	4
Proteins for translation	ATP-dependant protease	*clpP* **	1
	Translation initiation factor A	*infA*	1
	Ribosomal protein (large)	*rpl2* * *x2, rpl14, rpl16, rpl20, rpl22, rpl23* * *x2, rpl32,rpl33, rpl36*	10
	Ribosomal protein (small)	*rps2*, *rps3*, *rps4*, *rps7*x2, *rps8*, *rps11*, *rps12* **x2, *rps14*, *rps15*, *rps16* *, *rps18*, *rps19*	12
Structural RNAs	Ribosomal RNAs	*rRNA 4.5S*x2, *rRNA 5S*x2, *rRNA 16S*x2, *rRNA 23S*x2	4
	Transfer RNAs	*tRNA-His*(GUG), *tRNA-Lys*(UUU)*, *tRNA-Gln*(UUG), *tRNA-Ser*(GCU), *tRNA-Gly(*UCC)*, *tRNA-Arg*(UCU), *tRNA-Cys*(GCA), *tRNA-Asp*(GUC), *tRNA-Tyr*(GUA), *tRNA-Glu*(UUC), *tRNA-Thr*(GGU), *tRNA-Ser*(UGA), *tRNA-Gly*(UCC), *tRNA-fMet*(CAU), *tRNA-Ser*(GGA), *tRNA-Thr*(UGU), *tRNA-Leu*(UAA)*, *tRNA-Phe*(GAA), *tRNA-Val*(UAC)*, *tRNA-Met*(CAU), *tRNA-Trp*(CCA), *tRNA-Pro*(UGG), *tRNA-Ile*(CAU)x2, *tRNA-Leu*(CAA)x2, *tRNA-Val*(GAC)*x2, *tRNA-Ile*(GAU)*x2, *tRNA-Ala*(UGC)*x2, *tRNA-Arg*(ACG)x2, *tRNA-Asn*(GUU)x2, *tRNA-Leu*(AUG)	30
**Conserved reading frames**		*ycf1*x2, *ycf2*x2, *yfc15*x2, *ycf68*x2	4
			103

* gen with one intron;

** gene with two introns; crossed missing;

Ψ pseudogene.

The 103 genes are grouped by function.

Out of the 103 genes encoded in the *Najas flexilis* plastid, four correspond to ribosomal RNAs, 30 to transfer RNA, and 69 are protein-coding genes. Usually, nearly 80 protein-coding genes are present in the angiosperm plastid genome [38]. The lower number of functional protein-coding genes in *Najas flexilis* is exclusively caused by the lack of functionality of the *ndh* genes (Fig. 1). Small fragments of truncated *ndh* genes were detected in the LSC region (*ndhC*) and in the SSC (*ndhD*, *E* and *H*) while only *ndhB*, in the repeat regions, maintained the expected gene size. However, the non-functional role of this gene was clearly indicated by the presence of stop codons and indels that cause frame shifts.

### 2. *Najas flexilis* Plastid Genome Maintains a Quadripartite Structure

Comparison of overall genomic structure of the *Najas flexilis* plastid using Mauve alignment in a broad phylogenetic comparison (from *Amborella* to Asterales) showed that it is collinear with the plastid genome reported for the angiosperm *Amborella*, sister to all other extant angiosperms [39] or other monocots, such as *Acorus* [40], *Lemna* [27] or *Elodea* [35] (data not shown). The plastome of *N. flexilis* has not suffered structural rearrangements affecting gene order and overall homology aside from loss of the *ndh* genes. Additional analyses using different phylogenetic groups (i.e., Alismatales and other monocots) produced the same result (Fig. S1).

**Figure 2:**
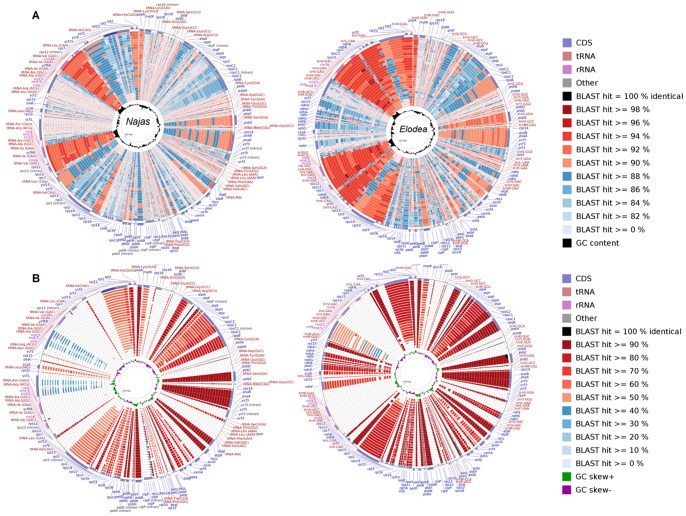
BLAST comparison of the *Najas flexilis* and *Elodea canadensis* chloroplast genomes. *Najas flexilis* (left) and *Elodea canadensis* (right) are compared to other monocots and selected species representing overall angiosperm diversity. The analyses were generated using blastn (**A**, DNA comparison) and blastp (**B**, CDS comparison) in CGViewer. In the upper part of the figure (**A**), schematic representation of the GC content is provided for each species. In the lower one (**B**), the values of GC Skew index are represented in green and purple. In each genome comparison, genes are color-coded by function. Blast hit values are color-coded by percentage of similarity. In the CDS analysis the bar height is proportional to the similarity value. For each figure, the specific order of the genome rings is determined by the similarity to the reference genome. This sorting emphasizes sequence divergence trends for CDS or sequences in the reference genome. Similarity was determined as defined in CGViewer, by a heuristic considering the total number of genome bases contributing to the hits and their scores [29]. Note: data for some included taxa were downloaded from Genbank: (in alphabetical order) *Acorus, Amborella, Calycanthus, Ceratophyllum, Chloranthus, Colocasia, Dioscorea, Drimys, Elaeis, Elodea, Fragaria, Illicium, Jacobea, Lemna, Magnolia, Najas, Nymphaea, Oryza, Phalaenopsis, Piper, Ranunculus, Smilax, Spirodela, Triticum, Wolffia, Wolfiella, and Zea.*

Comparison of the plastid genomes of *Najas flexilis* and *Elodea canadensis* reflected other shared structural features aside from gene collinearity. Both species shared equivalent distributional patterns of GC islands or areas where G and C are distributed unevenly between the DNA strains (Fig. 2). However, in a broader phylogenetic context, *Najas flexilis* is highly divergent. Blast analysis showed lower overall sequence identity to other angiosperms than did the closely related *Elodea canandensis,* when both were compared within the same broad phylogenetic representation (Fig. 2). Although *Elodea canadensis* presents 90% sequence similarity across the entire region, in *N. flexilis* this level of high similarity is restricted to the IR regions, ribosomal gene clusters, tRNAs and the junction area, where *rpl2* is encoded. In the *Najas* plastid, protein-coding sequences that show high similarity to other angiosperms are related to photosynthesis. Genes encoding Photosystem I and II, ATP synthase or RuBisCO are highly conserved both at the nucleotide and protein level. Some other genes are highly dissimilar, such as *clpP*, *ycf1* and *ycf2*.

**Figure 3:**
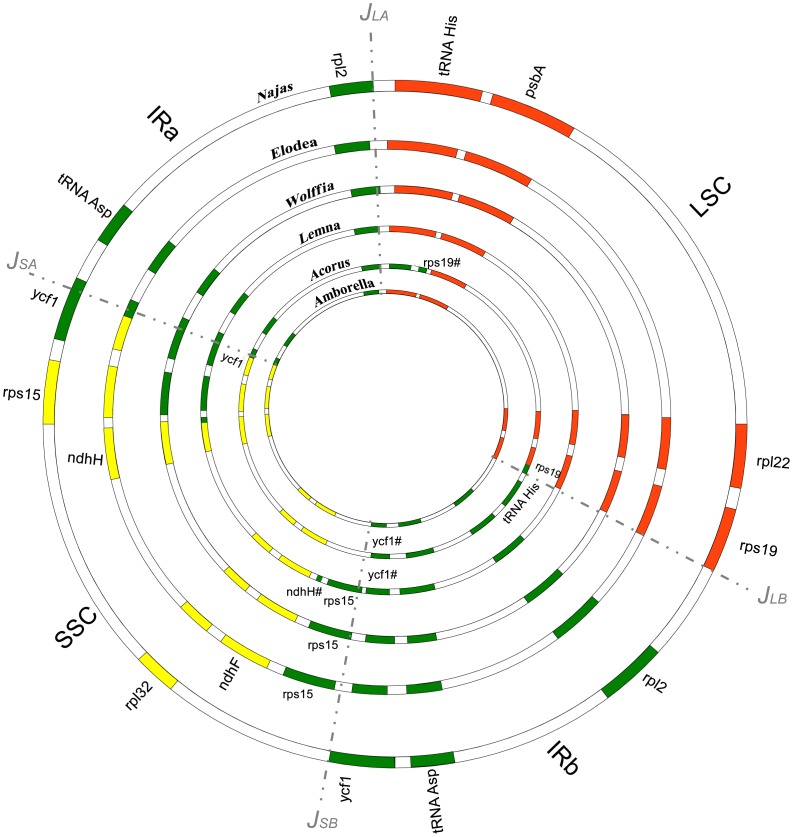
Comparison of expansion and contraction patterns that lead to junction changes between the IR and single copy regions (J_LA_, J_SA_, J_SB_, J_LB_). The chloroplast genome of *Najas flexilis* is compared to those available for Alismatales, *Acorus,* sister to all other monocots, and *Amborella,* sister to all other angiosperms. Genes involved in IR expansions are color-coded. Green, genes in the IR regions; orange, LSC; and yellow, SSC. Dashed lines indicate the expected junction area. #, truncated gene.

The quadripartite structure of the plastid genome is characterized by the existence of highly dynamic junctions between the IR and single copy regions (J_LA_, J_SA_, J_SB_, J_LB_). Events that cause the expansion and contraction of the IR regions account for most of the size variation among genomes of different taxa [41]. Size changes are caused by translation of the junctions and inclusion or exclusion of genes in the repeat regions. Plastid junctions have been suggested as evolutionary markers for elucidating relationships among taxa [41]. The plastid genome of *Najas flexilis* shares the general organization described for the Alismatales [27], [35], [37]. Most monocots, including *Acorus*, are characterized by the presence of a *trnH* gene in each repeat region [40]. The J_LA_ junction is situated downstream of the start codon of *rps19*, producing either a truncated or complete *rps19* gene in the IRs. Analysis of the interspacer region of the *trnH*-*rps19* cluster has suggested that this feature evolved from a single event in monocots [41]. The order Alismatales differs from other monocots at the J_LA_ and J_LB_ areas, as the genes *rps19* and *trnH* are localized in the LSC region. This configuration is consistent with that reported for *Amborella* [39] or *Nymphea* [42], well supported sister taxa to all other angiosperms [34]. However, analysis of the intergenic spacers confirmed that these sequences in Alismatales are more similar to other monocots than to any other group of angiosperms and might have resulted from a separate contraction [40]. The J_LA_ and J_LB_ junctions in the *Najas flexilis* plastid present no differences to those previously reported for Alismatales (Fig. 3) [27], [35], [37].

On the other hand, the position of the SSC and the IR junctions (J_SA_ and J_SB_) differs within different families in the Alismatales. The family Araceae, including *Lemna* or *Wolffia*, is characterized by the inclusion of *rps15* in the IRs [27], [37] whereas *Najas* and *Elodea* (Hydrocharitaceae) maintain *rps15* in the SSC region, a shared trait with *Acorus* and *Amborella*, which are respectively sister to all other monocots, and all other angiosperms. The J_SA_ in *Elodea canadensis* is within *ycf1*, producing a truncated gene in the IR. In *Najas flexilis,* J*_SA_* is sited downstream of *rps15*, resulting in the presence of two full copies of *ycf1* in its plastid genome (Fig. 3).

### 3. *ndh* Genes are Lost in the *Najas flexilis* Plastid

Plastid genes can be ascribed to light-dependent or light-independent photosynthetic pathways, to non photosynthetic related functions and to the genetic apparatus [38]. The number of genes encoded in plastid genomes of photosynthetic plants typically is 100–120, nearly 80 of these genes are translated into proteins. Monocots present little over 110 genes (*Acorus* 112 [40]; *Lemna* 112 [27]; *Phoenix* 112 [43]; *Elodea* 113 [35]; *Hordeum*, *Sorghum* and *Agrostis* 113 [44]; *Smilax* 114 [36]). Usually, differences in the number of genes reported are related to the presence of the ORFs *ycf15* and *ycf68*. The function of these genes, both present in *Najas flexilis* (Table 1), still remains unclear, although they have been suggested to act as transcriptional regulators [38].

**Figure 4:**
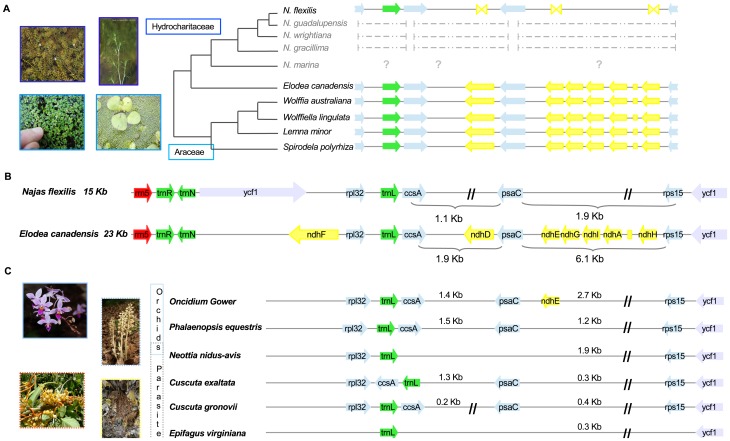
Loss of *ndh* genes in the small single copy region (SSC). The *ndh* genes are represented by yellow arrows or truncated yellow arrows; blue, protein-coding genes; green, tRNA; red, rRNA; grey, genes of unknown function. Parallel lines indicate areas reduced in size. **A.** Comparison of genes detected in the SSC regions in different Alismatales. The tree is a consensus of those published elsewhere and is included only to illustrate phylogenetic relationships among the species. All members of the family Araceae (light blue) possess a complete set of *ndh* genes. In the family Hydrocharitaceae (dark blue), the *ndh* genes are retained in *Elodea canadensis* but missing in *Najas*. The dashed lines represent sequences retrieved by targeted-PCR in other *Najas* species. **B.** Schema of the IR/SSC region in *Najas flexilis* and *Elodea canadensis*. **C.** SSC regions in orchids (blue) and parasitic plants (dashed blue, Orchidaceae; dashed orange, Solanales; dashed yellow, Lamiales). The ‘loss as a suite’ pattern in these groups is equivalent to the example reported here for *Najas flexilis*.

The *Najas flexilis* plastome encodes the complete set of structural ribosomal RNA genes and 30 transfer RNAs. All protein-coding genes related to transcription, translation and intron maturation are intact. While the groups of genes involved in synthesis of Photosystem I and II, ATP synthase, and the cytochrome b6/f complex are intact, none of the genes encoding the NAD(P)H dehydrogenase complex is functional (Table 1). Out of the 11 *ndh* genes in the plastome, six are missing (*ndhJ*, *ndhK*, *ndhF*, *ndhG*, *ndhI*, *ndhA*) and only pseudogenized or truncated sequences of the other five can be found (*ndhCΨ*, *ndhBΨx2*, *ndhDΨ*, *ndhEΨ*, *ndhHΨ*) in *Najas flexilis*. The loss or pseudogenization of the *ndh* genes is responsible for the small size of the SSC (Fig. 4B) region (5 266), which is less than one third of that reported for *Elodea canadensis* (17 810 bp) [35]. In this study, the fate of the nuclear-encoded *ndh* genes *ndhL*, *ndhM*, *ndhN*, and *ndhO* is not explored.

To confirm the lack of *ndh* genes in the plastid genome of *Najas flexilis*, specific primers were designed surrounding predicted areas for gene encoding (Table 2). PCR and sequencing of the amplified fragments in *Najas flexilis* confirmed in each case partial or total loss of *ndh* genes. Amplified fragment size corresponding to the LSC region, where *ndhJ*-*C* genes are encoded, was one third shorter than expected by comparison with the same region in the *Elodea* plastid genome. Only 159 nts were identified as part of a truncated *ndhC* (*Elodea*, *Lemna* 363 nt), while total loss of the *ndhJ* and *ndhI* genes was confirmed. Similar results were confirmed for the SSC region, most notable being a *psaC*-*rps15* reduction (Fig. 4A). General gene organization in this area is highly conserved across the green plant phylogeny. It usually expands over 6 kb encoding the *ndhH*-*D* operon, including six *ndh* genes and *psaC*. In *Najas flexilis*, *psaC* is just 1.9 kb away from *rps15*, while in *Elodea* this distance is 6.1 kb (Fig. 4B). This size reduction is caused by alteration of the *ndh* genes in the *ndhH*-*D* operon where only truncated *ndhH*, *ndhE* and *ndhD* genes were detected. Not even small fragments of the three other *ndh* genes (*ndhA*, *ndhI*, and *ndhG*) were detected by DOGMA.

**Table 2:** Primer sets to amplify the regions containing *ndh* genes.

Chloroplast region	Primer name	Gene		Sequence	TM	Position	Product size(*Elodea*)	Product size (*Najas*)	Missing genes (*Najas*)
LSC-Leu	*tRNA-Leu*-1F	*tRNA-Leu*(UAA)	Forward	TACACGGCCAAGGAATCTCG	59.8	51740	>2400	831	*ndhJ*, *ndhK*, *ndhC*Ψ
	*tRNA-Leu*-1C-F	*tRNA-Phe*(GAA)	Forward	AGGGTCGAGTCAGGATAGCT	59.4	51960	>2100	611	
	*tRNA-Leu*-1R	*ndhC*	Reverse	GGGTATTTGTCACCGGGGTT	60	52570			
IR b_SSC	NF_IRb_SSC_3F	past *ycf1*	Forward	CTTCGGAAGAAATCCATTGGGCAAAAA	57.02	119590	?	324	*ndhF*
	NF_IRb_SSC_3R	*rpl32*	Reverse	TGGAACTGCCATTTCAAAGCGACT	57.58	119914			
SSC_*rpl32*	NF_SSC_*rpl32*_1F	*rpl32*	Forward	TTGGCTGCGGTTAAAGCTTTTTCTTT	57.02	119977	>1350	1269	none
	NF_SSC_*rpl32*_1R	*ccsA*	Reverse	CCACTGCGACTGTAGAGCAGGC	59.55	121246			
SSC_*ccsA*	NF_SSC_*ccsA*_1F	*ccsA*	Forward	GCCTGCTCTACAGTCGCAGTGG	59.55	121225	>2400	1688	*ndhD*Ψ
	NF_SSC_*ccsA*_1R	*psaC*	Reverse	TGCTTCCGCGCCAAGAACAGA	59.32	122913			
SSC_*psaC*	NF_SSC_*psaC*_1F	*psaC*	Forward	GTCCTCTGTTCTTGGCGCGGA	59.13	122889	>6400	1763	*ndhE*Ψ, *ndhG*, *ndhI*, *ndhA*, *ndhH*Ψ
	NF_SSC_*psaC*_1R	*rps15*	Reverse	CGAAGGATTTTAGGAAAACGCCAACG	57.58	124652			

Ψ pseudogene.

Primers are transferable among different *Najas* species.

Loss of the *ndh* genes is the main cause of the extremely small size of the SSC region across the *Najas* genus (Fig. 4A). Successful amplification was achieved in other *Najas* species using the same primer sets described for *N. flexilis* (Table 2). Unfortunately, due to high divergence, amplification of the SSC region was not possible in *N. marina,* the only species in the subgenus *Najas* (see [23] for a revised phylogeny of the genus *Najas*). PCR amplification and sequencing of the SSC region in other *Najas* species, such as *N. guadalupensis, N. wrightiana* (section *Americanae*) and *N. gracillima* (section *Euvaginatae*), indicate that loss of *ndh* genes and a reduced SSC are traits shared across the genus *Najas*. The size of amplified fragments in other *Najas* species matches that reported above for the *ndh*-defective *Najas flexilis.* Alignment of the sequences and comparison with the *Najas flexilis* plastid genome allowed the unequivocal identification of the fragments as intergenic regions in the SSC (Fig. 4A). In each case, the reduced size of the amplified fragments and posterior sequence analysis strongly suggest loss of the plastome *ndh* genes across the *Najas* genus and the maintenance of non-*ndh* genes in the region. Loss of *ndh* genes in all *Najas* species probably follows the ‘loss as a suite’ pattern reported for Pinaceae, Gnetales, orchids and parasitic species [8]–[17], [20] (Fig. 4C).

Loss of *ndh* genes in the genus *Erodium* (Gerananiaceae) has been defined as the most recent (5–15.45 MYA) and phylogenetically restricted amongst photosynthetic seed plants [21]. This loss was reported only in species belonging to the ‘long-branch clade’ (LBC) while complete plastid genome sequences of other *Erodium* species confirmed the presence of functional *ndh* genes in other clades. In the Alismatales, loss of the *ndh* genes is a homoplastic feature. PCR-targeted analysis detected alterations in *ndhF* and *ndhB* in seagrasses of the tepaloid clade (*Posidonia* and *Amphibiolis*) and in members of the petaloid clade (*Najas flexilis* and *Thalassia)* suggesting that loss of *ndh* genes has occurred several times in the Alismatales [18]. Sequencing of the *Najas flexilis* plastome confirms that the lack of amplification of *ndhF* in *Najas* was caused by the complete loss of the gene. Maximum Likelihood tree (data not shown) of the *ndhB* gene successfully resolve the Hydrocharitaceae family with an equivalent topology to that obtained with 17 plastid genes [18]. *Najas* and *Thalassia* pseudogenized *ndhB* genes share a common origin with those functional *ndhB* genes in the *Elodea canadensis* (and possibly *Hydrocharis* and *Stradiotes*) plastome. The pseudogenization of *ndhB* in the petaloid clade is independent of the event in the *Posidonia*-*Amphibiolis* clade (tepaloid).

### 4. Implications of the Loss of the NDH Complex in Aquatic Angiosperms

The plastome *ndh* genes, equivalent to those present in the mitochondria, encode the NAD(P)H dehydrogenase complex located in the stromal thylakoid. The main function of this complex is electron transfer from NADH to plastoquinone, and plays an essential role in land plant photosynthesis by protecting against photooxidative-related stress and maintaining optimal rates of cyclic photophosphorylation [5]. Strong selection acting on the *ndh* genes would explain the high conservation evident across large phylogenetic distances, even including the angiosperm-gymnosperm divergence [7], [8]. Previously, *ndh* genes have been reported as rich in editing sites [45], [46]. Although post-trancriptional editing in chloroplasts is not fully understood, it is evident that it is a highly specific process [47]. Under controlled conditions, editing of most of the C targets might not be essential for plant survival [reviewed in [48]. It can be argued however that, in less optimal conditions, individuals capable of altering their transcripts to conserved amino acids might present a selective advantage [47]. Functional characterization of the NDH complex has shown that even a single amino acid change in one of the genes can decrease net photosynthesis by up to 20% under field conditions but has no effect under milder conditions (e.g. fluorescent light) [6]. This lack of effect under certain environmental conditions might provide an explanation for the accumulation of editing sites in the *ndh* genes of flowering plants. RNA editing mechanisms would have allowed the rescue of *ndh* genes after episodes of dispensability during plant evolution [5].

In Cyanobacteria, *ndh* genes may encode several complexes formed by different NDH subunits. It has been suggested that these complexes could have different genetic regulation and different functions such as cyclic electron transport in Photosystem I, respiration and CO_2_ uptake [5]. As an endosymbiont derived from a free-living cyanobacterium, the plastid genome has suffered gene reduction and has readjusted its functions during the evolution of photosynthetic organisms [38]. Some of the forces driving those changes have been related to land colonization with photosynthetic organisms needing to adapt to new environments characterized by high light intensities. These new conditions forced the photosynthetic-related genes to acquire new roles as a means of coping with fluctuating conditions of the terrestrial environment. The *ndh* genes may have acquired new functions to improve photosynthetic performance under rapidly fluctuating terrestrial conditions [5]. Most of the algae (red, golden, brown) have lost the *ndh* genes. Excluding the exceptional case of the genus *Nephroselmis*, only the Charophyte algae, which include the presumed ancestor of land plants (Embryophytes) and usually inhabit waters of fluctuating shoreline environments, maintain the set of *ndh* genes [5].

During the evolution of land plants, aquatic environments have been re-colonized repeatedly. Aquatic plants are presently represented in roughly 17% of angiosperm families as the result of 100 independent evolutionary origins [22]. Adaptation to the aquatic environment is a difficult task and it involves major changes in morphology, physiology, and even reproductive strategies. Many aquatic species deal with water-associated problems of low light intensity or pollination by performing some of their functions on the surface, using floating or emergent leaves and flowers. Few species of angiosperms, however, have been able to solve all associated problems to enable a return to the completely submersed state [22]. For angiosperms, colonization of the aquatic environment requires the acquisition of traits equivalent to those present in algae, such as underwater reproduction. At the same time, selective forces to retain features specifically tied to terrestrial habitation disappear. While in land plants a completely functional *ndh* complex might provide adaptive advantages to deal with fluctuating light-intensities [6], submersed aquatics have to re-adapt their entire photosynthetic apparatus to low light conditions [18], [19]. The *Najas flexilis* plastid genome indicates that the relaxed pressure on some photosynthetic genes, as a consequence of the return to the aquatic environment, can lead to extreme genetic modifications or losses. As in deep water algae, the NDH complex, once essential for land-colonization, appears to be dispensable in at least some submersed angiosperms like *Najas*. Further studies of aquatic plant chloroplast genomes should determine whether the loss of these genes is characteristic of hydrophytes in general.

## Supporting Information

Figure S1 **Mauve alignment of the chloroplast genome structures among the order Alismatales using** ***Acorus americanus*** **as the reference.** The color-coded boxes represent genome segments. In the diagram the lower boxes represent the genes transcribed in reverse direction. Genes are color-coded: white, protein coding genes; green, tRNA; red rRNA; pink boxes, IR. Numbers above the boxes indicate nucleotide positions from the origin.
(TIF)

Table S1 **Primer sets used in the chloroplast genome of** ***Najas flexilis*** **for amplification and Sanger sequencing of assembly gaps, low coverage areas and junctions.**
(XLS)
