# Supplementary figures and images for: The Plastid Genome of Najas flexilis: Adaptation to Submersed Environments Is Accompanied by the Complete Loss of the NDH Complex in an Aquatic Angiosperm
Source: PLoS One. 2013 Jul 4;8(7):e68591. doi: 10.1371/journal.pone.0068591 (PMC3701688; doi:10.1371/journal.pone.0068591)

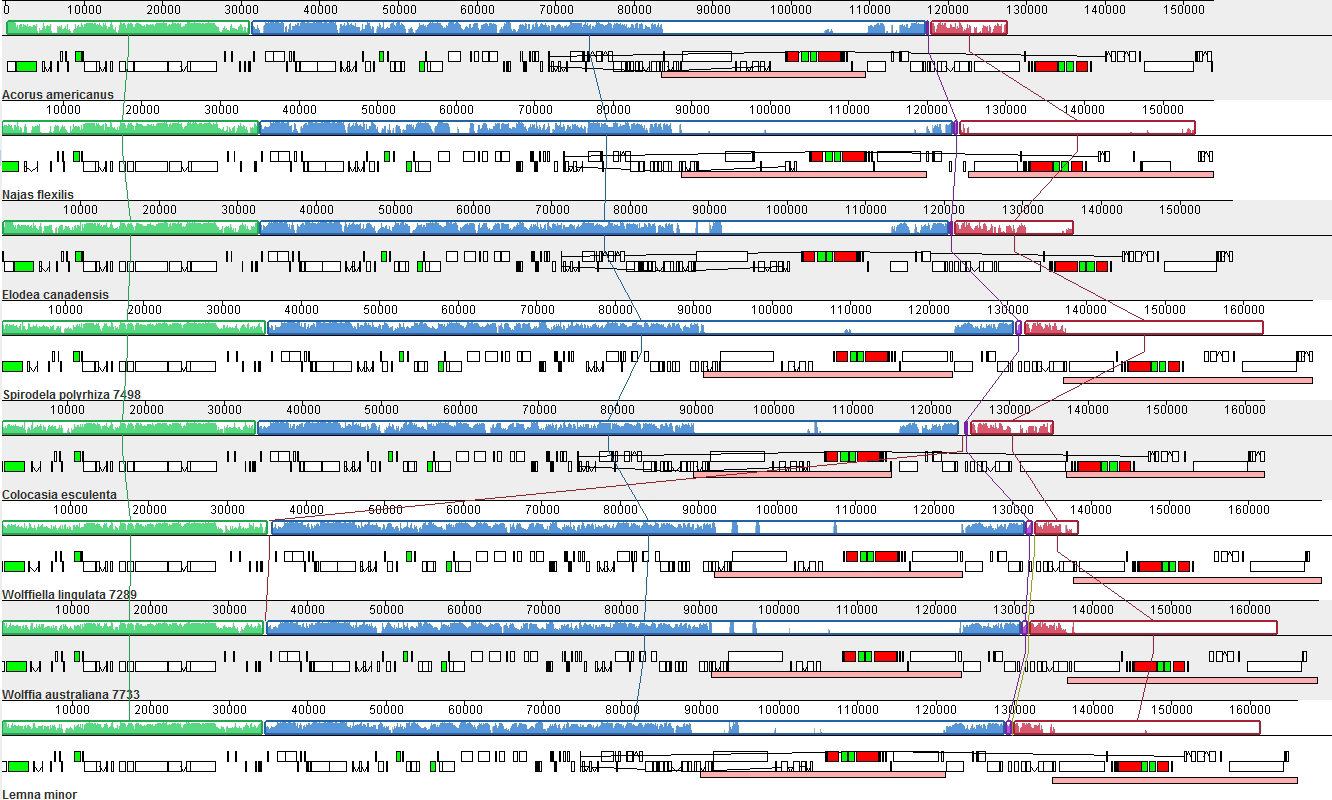

Supplement: Figure S1 — Mauve alignment of the chloroplast genome structures among the order Alismatales using Acorus americanus as the reference. The color-coded boxes represent genome segments. In the diagram the lower boxes represent the genes transcribed in reverse direction. Genes are color-coded: white, protein coding genes; green, tRNA; red rRNA; pink boxes, IR. Numbers above the boxes indicate nucleotide positions from the origin. (TIF) [file pone.0068591.s001.tif]
